# Cannabinoids for the treatment of refractory neuropathic pruritus in amyotrophic lateral sclerosis: A case report

**DOI:** 10.1177/02692163211045314

**Published:** 2021-09-11

**Authors:** Kelvin Lou, Shane Murphy, Clair Talbot

**Affiliations:** Palliative Medicine, The University of British Columbia, Vancouver, BC, Canada

**Keywords:** Cannabis, cannabinoids, small fiber neuropathy, amyotrophic lateral sclerosis

## Abstract

**Background:**

Neuropathic symptoms have a wide variety of manifestations, ranging from pain to pruritus. Neuropathic pruritus is a type of chronic pruritus related to damaged small fibers. Cannabinoids have evidence to manage neuropathic symptoms. We present a case of refractory neuropathic pruritus that was successfully managed with the use of oral cannabinoids.

**Case presentation:**

A 60-year-old male with amyotrophic lateral sclerosis with ongoing pruritus despite the use of standard neuropathic therapies.

**Possible course of action:**

Sodium channel and *N*-methyl-D-aspartate receptor antagonists have evidence for neuropathic symptoms but can cause significant gastrointestinal side effects. Prescription cannabinoids such as nabiximol can be cost prohibitive to use in practice. Synthetic tetrahydrocannabinol products are dose limited by psychoactive side effects.

**Formulation of a plan:**

A balanced oral cannabinoid from a licensed producer was preferred as it has evidence for neuropathic symptoms and is generally well tolerated.

**Outcome:**

The patient showed improvement to his pruritus score from 7/10 to 3/10. There was initial increased sedation but tolerance developed quickly.

**Lessons learned from case:**

Cannabinoids are possibly safe and effective in management of neuropathic pruritus.

**View on research problems:**

Additional research is needed to establish efficacy and safety.

**What is already known about this topic?**

• Neuropathic pruritus is a chronic form of pruritus that causes significant symptom burden and can be difficult to treat. Cannabinoids have evidence to manage chronic neuropathic pain.

**What this paper adds?**

• This case demonstrates the safe and effective use of cannabinoids to manage neuropathic pruritus.

**Implications for practice, theory, or policy**

• This report show a potential role for cannabinoids for the management of neuropathic symptoms and highlights the need for further clinical trials.

## Background

The clinical manifestations of neuropathic symptoms can range from pain, numbness, paraesthesia, or pruritus.^1^ Neuropathic pruritus is a type of chronic pruritus caused by the dysfunction of itch-sensing neurons and damage to the C-fibers and A-delta fibers.^1,2^ Amyotrophic lateral sclerosis is a disorder characterized by the degeneration of upper and lower motor neurons leading to diffuse weakness and muscle wasting. There is growing evidence that amyotrophic lateral sclerosis is associated with small fiber neuron loss, which can be a cause of neuropathic pruritus.^2,3^ The presentation of neuropathic pruritus has a large overlap with other causes of pruritus, but can include sensations such as pain, burning, tingling, or numbness in a distal-to-proximal pattern.^1^ Management of neuropathic pruritus begins with targeted investigations to look for reversible causes such as poorly controlled diabetes, thyroid disorders, or autoimmune conditions. The work up can also include nerve conduction studies and labs to rule out metabolic derangements such as uremia or hyperbilirubinemia.^2^ Chronic pruritus can be difficult to control and these symptoms can lead to decreased quality of life.^1^ The first-line treatments for non-cancer neuropathic symptoms include antidepressants and gabapentinoids.^4^ Topical agents such as moisturizers, capsaicin and menthol also serve as useful adjuncts.^4^ Given the lack of long-term efficacy of opioids in chronic non-cancer neuropathic pain, they are generally not recommended as first-line agents by international guidelines.^4,5^ As an alternative to standard therapies, there is emerging evidence for the use of cannabinoid within palliative care.^4–6^ The pharmacologically active components of cannabinoids include tetrahydrocannabinol (THC) and cannabidiol (CBD), which work as agonists on the endocannabinoid system.^7^ The endocannabinoid system is a homeostatic regulator that is involved with many physiological function including neurotransmitter regulation, stress response, immune function, and nociceptive processing.^7^ Tetrahydrocannabinol and cannabidiol work together synergistically and there is evidence that presence of cannabidiol may improve the tolerability of the psychoactive side effects of tetrahydrocannabinol.^7^ Synthetic tetrahydrocannabinol products without cannabidiol such as nabilone are often limited by psychoactive side effects.^7^ Balanced formulations such as nabiximol with both tetrahydrocannabinol and cannabidiol may be better tolerated as the CBD component helps mitigate the psychoactive side effects.^6^

The evidence for cannabinoids is limited by small sample size, methodological design flaws, and lack of standardization of formulation and doses.^6^ There are numerus examples in the literature where epidemiological and retrospective findings have not been borne out in trial conditions.^8^ The strongest evidence comes from new trials with better trial design and stricter standardization of doses using pharmaceutical grade formulations. Based on international guidelines and multiple systematic reviews, cannabis has evidence for use in chemotherapy induced nausea and vomiting, multiple sclerosis spasticity, and intractable seizures in Dravet and Lennox-Gastaut syndromes.^5,7–9^ There is also growing evidence for the use of cannabinoids in a wide range of neuropathic pain conditions, such as diabetic, HIV, and multiple sclerosis.^4–6^ Although prescription cannabinoid products such as nabiximol are better studied, their use is limited by significant cost and lack of third-party drug coverage. With the legalization of medical cannabis in various parts of the world, patients are able to access medical cannabis products through avenues such as Licensed Producers.^10^ Depending on the jurisdiction, Licensed Producers or their equivalents, are federally licensed distributors that undergo strict monitoring to ensure product quality and safety.^10^

## Case presentation

We present the case of a 60-year old male with a history of amyotrophic lateral sclerosis and chronic neuropathic pruritus. The medical history is also significant for gastroparesis and constipation despite regular laxatives. He is followed by the outpatient Palliative Care Team in the community for symptom management. In regards to his amyotrophic lateral sclerosis, he is managed with 24-h nasal Bilevel Positive Airway Pressure and essentially has no use of his arms or hands. One of the persistent symptoms he has had over the years is chronic pruritus. Originally, the pruritus was intermittent and only involved the tips of the extremities. Over the years, it has become progressively worse where it is now a constant generalized pruritus involving the entire body. These symptoms persisted despite good adherence to topical moisturizers and menthol ointments. More recently in the last year the pruritus is now associated with paraesthesia, autonomic symptoms, and temperature changes along the skin of the chest. Given his minimal upper limb function, he is unable to scratch the affected areas and relies on his caretakers to provide relief. The constant nature of this symptom, especially at night when there is no one to provide scratching has caused significant distress and has interfered with his sleep and mood. Investigations revealed vitamin B12 256 pmol/L, Thyroid stimulating hormone (TSH) 1.01 ng/dL, and A1C 5.7%. Renal, liver enzymes, and protein electrophoresis were within normal range. The ANA and extractable nuclear antibody panel were both negative. Non-pharmacological strategies trialed include using an alternating pressure mattress and various topical moisturizers. Other topical therapies trialed include menthol and camphor ointment, steroid cream, antihistamine cream, and capsaicin cream. Systemic therapies trialed include hydroxyzine 25 mg three times daily, pregabalin 50 mg twice daily, venlafaxine 150 mg daily, nortriptyline 10 mg, and mirtazapine 15 mg. Each therapy was only helpful for a short time and the doses of systemic therapy were limited by significant sedation and anticholinergic side effects.

**Possible course of action:** Medications with alternative mechanisms of action include sodium channel blockers and *N*-methyl-D-aspartate receptor such as mexiletine and methadone respectively. Both classes of medication have evidence for neuropathic pain, but the significant gastrointestinal symptoms the patient has at baseline precludes their use.^2^ Psychostimulants have been used to manage medication related sedation but this would contribute to polypharmacy and the long-term safety is unclear.^11^ The use of prescription cannabinoid products such as nabiximol is limited by the significant out of pocket costs. Synthetic tetrahydrocannabinol products such as nabilone are often dose-limited by psychoactive side effects.

## Formulation of a plan

Given the unresolving symptoms, a discussion was had regarding alternative treatment options, including antiepileptics, sodium channel blockers, or cannabis. After reviewing the risks and benefits of each, the patient decided to trial cannabinoid products from a federally licensed dispensary. The cannabinoid product chosen was a balanced oral capsule formulation (2.43 mg THC/CBD 2.75 mg). For pruritus intensity assessment, the numeric rating scale, which ranges from 0 (no pruritus) to 10 (worst possible pruritus) was used. At baseline evaluation, he was maintained on pregabalin 50 mg twice daily.

## Outcome

Prior to the administration of the cannabinoid capsule, his pruritus score was 7/10 at baseline. He was started initially on one capsule daily, which improved his pruritus score to 5/10 after 2 days. During the first 2 days, he reported mild sedation up to a few hours after medication administration. By the third day, he had developed tolerance to the sedation and the dose was increased to one capsule twice daily. At the twice daily dose, his pruritus score decreased to 3/10 within a few days. Given the marked improvement to his pruritus, he was weaned off pregabalin over the course of 2 weeks with no change to his pruritus score and there was improvement to his level of sedation. During subsequent follow-up visits, the patient reported that the level of pruritus control is sufficient and that the improvement has been sustained. During the treatment course with cannabis, there was no change in the levels of anxiety experience by the patient but he did experience an overall improvement to his wellbeing given the improved symptom control.

## Lessons learned from the case

This case report describes a sustained reduction of generalized pruritus of potential neuropathic origin by oral cannabinoids in a patient with ALS. Although there was mild sedation initially, tolerance developed quickly. The sustained efficacy is further supported by the weaning of pregabalin without worsening of pruritus control. Shared decision-making with the patient allows for additional treatment options when standard therapies are exhausted. With the increased availability and improved regulation of cannabinoid products, it may prove to be a useful tool to manage symptoms in the future.

**View on research problems, objectives or questions generated by the case:** This case highlights the need for additional studies such as prospective-case series and placebo-controlled studies to establish efficacy and safety of cannabinoids.
